# The Role of Heavy Metals in Plant Response to Biotic Stress

**DOI:** 10.3390/molecules23092320

**Published:** 2018-09-11

**Authors:** Iwona Morkunas, Agnieszka Woźniak, Van Chung Mai, Renata Rucińska-Sobkowiak, Philippe Jeandet

**Affiliations:** 1 Department of Plant Physiology, Poznań University of Life Sciences, Wołyńska 35, 60-637 Poznań, Poland; agnieszkam.wozniak@gmail.com (A.W.); chungmv@vinhuni.edu.vn (V.C.M.); 2 Department of Plant Physiology, Vinh University, Le Duan 182, Vinh City, Vietnam; 3 Department of Plant Ecophysiology, Adam Mickiewicz University, Umultowska 89, 61-614 Poznań, Poland; renatar@amu.edu.pl; 4 Research Unit “Induced Resistance and Plant Bioprotection”, UPRES EA 4707, Department of Biology and Biochemistry, Faculty of Sciences, University of Reims, P.O. Box 1039, 02 51687 Reims CEDEX, France; philippe.jeandet@univ-reims.fr

**Keywords:** hormesis, toxic effect, heavy metals, biotic stressors, cross-talk, plant defence responses

## Abstract

The present review discusses the impact of heavy metals on the growth of plants at different concentrations, paying particular attention to the hormesis effect. Within the past decade, study of the hormesis phenomenon has generated considerable interest because it was considered not only in the framework of plant growth stimulation but also as an adaptive response of plants to a low level of stress which in turn can play an important role in their responses to other stress factors. In this review, we focused on the defence mechanisms of plants as a response to different metal ion doses and during the crosstalk between metal ions and biotic stressors such as insects and pathogenic fungi. Issues relating to metal ion acquisition and ion homeostasis that may be essential for the survival of plants, pathogens and herbivores competing in the same environment were highlighted. Besides, the influence of heavy metals on insects, especially aphids and pathogenic fungi, was shown. Our intention was also to shed light on the relationship between heavy metals deposition in the environment and ecological communities formed under a strong selective pressure.

## 1. Introduction

Heavy metals belong to the group of the most dangerous pollutants in ecotoxicology regarding their high toxicity and their significant amounts released into the environment as a result of natural and anthropogenic processes [1,2,3,4]. This situation creates a need for research focusing on the assessment of the impact of heavy metals on living organisms and other elements of the natural environment. Heavy metals might be poisonous depending on the metal type, its oxidation state, pH, concentration, duration and so forth, leading to the death of organisms. The toxicity of a specific substance depends on a variety of factors, including how much of the substance organisms are exposed to, how they are exposed and for how long. It is documented that chemicals at a low dose may have beneficial effects that is termed hormesis and at a high dose, harmful effects. In this review, we focus on the impact of heavy metals at hormetic and toxic doses on plant responses and how their presence in the surrounding environment affects the response of plants to biotic stressors such as insects, especially aphids and pathogenic fungi playing a definite role in the trophic chain (Table S1 and Table 1). Additionally, the effect of heavy metals on insects, especially aphids and fungi were demonstrated (Table 2 and Table 3). It has been documented that air and soil pollution, including heavy metals can affect the plants-insects/plants-pathogens relationships directly or indirectly, can be toxic or cause hormesis effect and change their behaviour and metabolism [5,6,7].

Understanding the mechanisms underlying plant resistance or tolerance of plants to abiotic and biotic stress factors is extremely important in the era of global warming, where the mobility of pollutants in the environment increases. Because plants are prone to various abiotic and biotic stresses, exposure of plants to one stress affects their response during the next stress leading to enhanced defence mechanisms to a later stress. This phenomenon called as “priming” results in a faster and stronger induction of basal defence mechanisms upon subsequent biotic stress factors [8]. This is an example of the occurrence of “metabolic memory” in higher plants [9,10], some stress “memory” or “stress imprinting” defined as genetic or biochemical modifications [11]. Moreover, it requires less energy expenditure than defence directly induced by insect feeding or infection caused by pathogens. Ahmad et al. [12] reported that priming-inducing stimuli can provide more effective basal resistance, especially when an earlier defence response precedes immune suppression by the invading pathogen. Convergence points in abiotic and biotic stress signalling networks were also shown [13]. On the other hand, the extremely interesting hypotheses of Poschenrieder and co-workers [14,15] such as elemental defence hypothesis, trade-off hypothesis, metal therapy and metal-induced fortification showed that metal ions may be factors that induce chemical defences against herbivores and infective microorganisms. Suitable intracellular concentrations of essential metal ions are not only required for the optimal growth and development of plants and their enemies but also for pathogen virulence and plant defence. In the presence of different toxic levels of metal ions, the degree of metal avoidance and/or tolerance of the interacting organisms will determine the response of plant host-insect/plant host-pathogen.

Of all the known elements, more than 75% exhibit the properties of metals. The metals were divided into two groups due to their density (specific atomic mass). Thus, light metals present a density of less than 5 g/cm^3^ and heavy metals are defined as elements having specific weights higher than 5 g/cm^3^ [16]. A number of them (e.g., cobalt-Co, iron-Fe, manganese-Mn, molybdenum-Mo, nickel-Ni, zinc-Zn, copper-Cu) are essential micronutrients required for normal growth and take part in redox reactions, electron transfers and other important metabolic processes in plants, whereas, others such as lead-Pb, cadmium-Cd, chromium-Cr, mercury-Hg, arsenic-As and so forth are potentially highly toxic for plants [17,18]. Heavy metals in the environment have various origins: they arise from natural processes such as rock weathering, volcanic eruptions, forest fires, soil-forming processes. However, the most important sources are anthropogenic processes. Large areas of land are contaminated with heavy metals resulting from mines, industries, urban activities and agricultural practices [19,20]. In terrestrial ecosystems, heavy metals mainly enter into the plants from soil but they may also originate from the external atmosphere surrounding plants [21]. One of the most serious problems related to metal toxicology is the contamination of soils with heavy metals, because they exhibit very high stability in soils and lack of biodegradability [22]. Therefore, metals present in soils can easily enter into the food chain [23]. According to the United States Environmental Protection Agency (UEPA), heavy metals are listed as priority pollutants. In terms of environmental risks, Pb, Hg, As and Cd are ranked as the first, second, third and sixth, respectively, in the list of US Agency for Toxic Substances and Disease Registry (ATSDR) [22]. 

The effects of metals in soils are very complex and these effects are dependent on chemical processes such as adsorption–desorption, complexation–dissociation, oxidation–reduction, ion exchange and transporters. Adsorption–desorption and complexation–dissociation reactions mainly affect metal activity in soil, while oxidation–reduction can also change metal valence [24]. The bioavailability and toxicity of metals in soils are significantly influenced by pH conditions [2,25]. Excessive concentrations of heavy metals inhibit physiological processes such as photosynthesis, respiration, transpiration rates, N-metabolism and mineral nutrition, cell elongation, decrease in biomass and consequently can cause plant death [26]. Plant responses to heavy metals are observed as changes in redox status, the level of signal molecules, the activity of antioxidant system enzymes, membrane permeability, cysteine content, glutathione (GSH) and phytochelatin (PCs) contents, protein content, expression of genes encoding pathogenesis-related (PR) proteins and genes encoding enzymes of the flavonoid biosynthesis pathway, level of phenolics and so forth (see references in Table S1). The impact of heavy metals is strongly related to their doses, the plant species and the plant developmental phase as well as environmental factors characteristic for a given climate zone. Toxic effects of metals on plants are generally well documented in the literature [27,28,29,30,31,32,33,34,35,36,37] but research on the responses of organisms, including plants, insects and fungal pathogens to heavy metals at the hormetic doses are less known and at the same time extremely interesting [38,39]. 

## 2. Hormesis as A Biological Phenomenon in the Context of Organisms’ Defence against Heavy Metals

In the published literature, the impact of heavy metals on the reduction of plant growth and modification of root morphology was widely presented [35,40,41,42,43]. Inhibition of the growth of plants under the effect of heavy metals leads to a decrease in yield, which is associated with the inhibition of most of the basic life processes of plants. However, at a low metal dose in the substrate, the opposite reaction can be observed [44]. In the present review, we focus on the progress of research into hormetic responses in terms of growth of plants, insects and fungi. The hormesis phenomenon has been observed in many organisms in response to different stress factors, that is, chemical, physical and biological. As mentioned above, already in the 16th century, Paracelsus wrote that various toxic substances may be beneficial in small quantities [45]. Calabrese and Baldwin [46] reported that the concept of chemical hormesis originate over a century ago from the research of Schulz [47], who observed a stimulation of both the growth and the respiration of yeast at low metal doses. Later, this concept was supported by other studies known as the Arndt-Schulz law. Moreover, in the opinion of some authors [46], hormetic effects are observed not only for growth but also during development, reproduction, survival and longevity. These authors reported that hormetic effects may also be closely related to numerous metabolic and physiological responses such as metallothionein synthesis, DNA and RNA syntheses, mitosis, oxygen consumption, photosynthesis rate, tissue regeneration, immune response, stress protein synthesis, seed germination and so forth. According to Damelin et al. [48], hormetic activity is a specific cellular response and most likely, a stress response to low but harmful levels of toxic agents. In another definition, hormesis refers to a process whereby a sublethal stressor renders an organism resistant to a subsequent stress. Additionally, an assessment of the toxicological basis of the hormetic dose-response relationship including implications for risk assessment, was provided by Calabrese [49]. In turn, a detailed assessment of the occurrence of hormesis within plants was also reported by Calabrese and Blain [50]. These authors presented the most prevalent chemical classes, including heavy metals and physical agents in the general hormesis database useful for plant studies. We summarized in Table S1, a list of various plant species, where the hormesis effect caused by various heavy metals has been observed. Additionally, information concerning the changes observed in various physiological and biochemical indicators of plants were also presented in Table S1.

For example, the hormesis effect of aluminium (Al) on the growth, development and biomass production of plants such as rice, tea, coffee, *Melastoma malabathricum*, soybean and maize was also recorded [44,51,52,53,54,55,56]. Moreover, numerous studies have reported the hormesis effect caused by Cd for plant species such as *Gypsophila fastigiata*, *Sedum alfredii*, *Viola baoshanensis*, *Lonicera japonica*, *Dianthus carhusianorum*, *Solanum melongena*, *Arabis paniculata*, *Brassica juncea*, *Salsola kali, Spirodela polyrrhiza, Arabis paniculata* [57,58,59,60,61,62,63,64,65,66]. This effect was also caused by As in the case of *Spirodela polyrrhiza* L. [65]. Besides, numerous experiments with chromium (Cr) revealed hormetic growth in the cases of *Eichhornia crassipes* [67], *Salsola kali* L. [68], *Lemna minuta* [69], *Allium cepa* L. [70] and *Capsicium annum* L. [71]. An hormetic response was observed as well, in the case of elements such as lanthanum (La) for *Lonicera japonica* [72], *Oryza sativa* L. [73] and *Arabis paniculata* [66], Pb for *Pisum sativum* [35], *Brassica pekinensis* [74] and *Dianthus carthusianorum* [60], Hg for *Anthyllis vulneraria* [75] and *Helianthus tuberosus* [76], palladium (Pd) for *P. sativum* L. [77], platinum (Pt) for *Arabidopsis thaliana* [78], uranium (U) for *Schizachyrium scoparium* [79], Zn for *Thlaspi caerulescens* [80], *Sedum alfredii* [57] and *Arabis paniculata* [66]. 

Additionally, an hormesis effect was also demonstrated in the case of insects. For example, hormesis as induced by Cd was reported in an insect model, such as the queen blowfly, *Phormia regina*, where low levels of dietary Cd caused a significant increase of the pupation rate of larvae [81]. Results of the above-mentioned research indicate the need to evaluate the impact of environmental stressors at different concentration ranges and during the entire life cycle. In turn, an hormetic effect was observed for insect survival of *Folsomia candida* under the influence of copper (Cu) [82]. Uranium (U) as well, caused hormetic responses in the case of earthworms (*Eisenia fetida*), which are the most abundant terrestrial species, playing an important role in maintaining the ecological function of soil [83]. *Eisenia andrei* (Oligochaeta: Lumbricidae), the potworm *Enchytraeus crypticus* (Oligochaeta: Enchytraeidae) and the springtail *Folsomia candida* (Collembola: Isotomidae) were also used as invertebrate test organisms in studying the hormesis effect in insects [83]. In turn, the glutathione *S*-transferase was involved in the induction of methylmercury (MeHg)-hormesis in the case of *Caenorhabditis elegans*, a free-living (non-parasitic) nematode [84]. These studies were performed to search whether MeHg induces hormesis, that is, whereby a sub lethal exposure to MeHg rendered *C. elegans* resistant to a subsequent exposure to this organometal. Previous studies of the same authors reported neurotoxic resistance of *C. elegans* to MeHg [85].

Nickel (Ni)-contamination of soils containing earthworms caused a hormesis response of the soil microorganisms that displayed a stimulation of microbial biomass C and soil basal respiration (SBR) [86]. Moreover, the hormesis effect has also been shown to accelerate pest population growth, insecticide resistance development, pesticide-induced arthropod pest resurgences, leading to interesting applications for the management of beneficial insects [87]. Environmental factors and other factors affect the amplitude of a wide range of hormetic responses [88]. For example, availability of nutrients, temperature, light and factors such as symbiosis, density-dependent factors, time, have an influence on hormetic responses.

In turn, a few studies reported hormetic responses to fungi. As shown by Calabrese and Baldwin [89], the assessment of the toxic effect of substances on fungi has a long history referring to the physiology, the growth and the reproduction of fungi. Already, Raulin [90] revealed that the sulphates of Zn and Fe markedly stimulated the growth of the fungus *Aspergillus* [46]. Raulin′s studies were aimed at showing quantitative relationships between disinfection agents and fungi. Establishment of fungal dose-response data near the turn of the 19th/20th centuries was carried out by investigators such as Stevens [91], Clark [92,93,94] and Duggar [95], who worked on the effect of numerous agents on fungal germination and/or mycelium growth and fruiting using wide metal dosage ranges. Later research, for example, Collin-Hansen et al. [96] demonstrated the toxic and the hormetic effects of the potential emissions from a Zn smelter to induce oxidative damages to DNA and lipids in *Boletus edulis*. These authors observed negative trends between the concentration of some metals (Cd, Zn, Cu and Hg) and apurinic/apyrimidinic sites or lipid hydroperoxides in the reference king bolete group. It has been indicated that in *B. edulis*, low concentrations of Hg, caused relationships of an hormetic nature such as an increase in metabolism, immunity and growth. Lefcort et al. [97] revealed that low levels of heavy metal pollution can have positive effects for populations of one snail species and one periphyton species. It should also be mentioned that a highly significant contribution to the history of hormesis was afforded by the research on yeast performed by Schulz [47].

## 3. Influence of Heavy Metals in the Response of Plants to Biotic Stressors and Cross-Talk between Heavy Metals and Biotic Stressors

Plants in the course of evolution demonstrate adaptation or tolerance to various abiotic and biotic stress factors. Responses of plants to environmental stresses involve constitutive defences as well as stress-inducible reactions. Stimulation of important tolerance pathways, such as enhancement of antioxidant enzymes activity, osmolyte accumulation, induction of membrane-localized transporters for efficient compartmentation of deleterious ions and accumulation of essential elements against pests or pathogens is also an area that has been intensively studied. Plants have developed mechanisms for ion homeostasis that enable them to cope with a certain limited excess of heavy metals [98]. Potentially toxic but essential metals required for normal growth have to be absorbed and distributed to different parts of the plant. Hall and Williams [99] reported that chaperons, chelators and specific transmembrane transporters have evolved. In plants non-adapted to given environmental conditions, that are challenged with ion excess, an enhanced biosynthesis of complexing substances such as phytochelatins, metallothioneins and/or organic acids can occur. It has been proven that in the case of arsenic (As) tolerance, normal constitutive tolerance as well as adaptive hypertolerance are based on phytochelatin-mediated sequestration [100,101]. In turn, in the case of copper (Cu) tolerance, it has been found that high Cu tolerance in a metallicolous population of *Silene vulgaris* was associated with high transcript levels of the metallothionein-like gene *SvMT2b*. Abundance and tissue distribution of efficient, specific, efflux and transport systems for metal ions and chelators seem fundamental traits for high-level tolerance in plants [102].

Some metal cations are also important for plant nutrition. Metals such as Cu, Fe, Zn and Mn act as important cofactors for many enzymes and are essential for both mitochondrial and chloroplast functions. However, when supplied in excess, these essential cations can become toxic like other heavy metals. To maintain micronutrient metal homeostasis and to cope with the deleterious effects of nonessential heavy metals, plants have developed a complex network of metal uptake, chelation, trafficking and storage processes. Metal transporters are required to maintain metal homeostasis in plant cells [20,99]. As reported by Ahanger et al. [103], plants sensitive to high levels of metals cannot survive in unfavourable environmental conditions, despite the induction of tolerance mechanisms. It has been documented that the plant responses to stress are dependent on the tissue or the organ affected by a given stress factor [104]. For example, transcriptional responses to stress are tissue or cell specific in roots and are quite different depending on the stress factor [105]. In addition, the level and the duration of the stress (acute vs. chronic) can have a significant effect on the complexity of the response. However, it should be noted that, in the environment, metals do not act as a single factor but simultaneous or subsequent effects of several other stress factors are observed. Here, our goal is to provide literature data evidencing phenomena such as cross-tolerance and cross-resistance in the case of heavy metal and biotic stress factor interactions [106,107,108,109]. Plant resistance mechanisms to heavy metals and biotic stress factors such as pathogenic fungi, bacteria and invertebrates, including insects are well known. However, progress in the field of abiotic and biotic stress signal transduction pathways and research results concerning metal hyperaccumulating plants make this topic still very interesting [106,110,111,112,113,114,115,116].

Responses to heavy metal excess in sensitive plants can resemble elicitor-induced defence reactions [117]. As an example of the negative cross-talk existing between abiotic stress (heavy metal) and biotic stress responses, the study by Llugany et al. [118] has indicated that Cd accumulation induces salicylic acid (SA) production in *Thymus praecox* but diminishes the plants′ ability to induce SA as a defence signal in response to attack by *Erysiphe cruciferarum*. Another study by Fones et al. [119] has shown that some pathogen-induced defences such as callose deposition and pathogenesis-related (PR) protein gene induction appears to be absent in *Noccaea caerulescens*, although the tested plants retained the ability to produce SA as a response to infection. This study also showed that the ability of *Pseudomonas syringae* to infect *N. caerulescens* plants grown on low concentrations of Zn was dependent on a functional type III secretion system (T3SS), a pathogenicity mechanism used by *P. syringae* to disable plant defences. This suggests that certain defence mechanisms that are suppressed by T3SS-secreted effector proteins remain functional in this plant. On the contrary, cross talk in down-stream signalling events has been well documented. As one of few candidates for such an early interaction, the cell wall associated kinase 1 (WAK1) is required for *Arabidopsis* resistance to *P. syringae*; plants over-expressing WAK1 exhibit enhanced Al tolerance [120]. Signal transduction pathways of biotic stresses offer multiple points of interaction with heavy metal stress signalling. Stress-activated increases in mitogen-activated protein kinases (MAPKs), phytohormones such as jasmonic acid (JA), salicylic acid (SA) and ethylene (ET) are in fact major cross points of interactions between pathogen-elicited responses and specific toxic effects of heavy metals. For example, exposure of *Medicago sativa* seedlings to excessive concentrations of Cu or Cd activates different MAPKs such as SIMK, MMK2, MMK3 and SAMK, thus suggesting that plants respond to heavy metal stress through the induction of several distinct MAPK pathways and that excess amounts of Cu and Cd induce different cellular signalling mechanisms [111]. Another screen for Cd-responsive genes identified the *Arabidopsis* MAPKKK MEKK1 to be transcriptionally induced by high concentrations of Cd [121]. JA is one of the typically phytohormones involved in signalling and induced as a result of cell damage by herbivores and necrotrophic pathogens but Cu and Cd were also found to induce JA in both *A. thaliana* and *Phaseolus coccineus* plants [122]. JA is connected to the toxic action of these two heavy metals in plants, differentially reacting to exogenous JA and possessing variable dynamics depending on the studied plants as well as their growth stage. The Ni tolerant capability mediating SA signalling pathway in *Thlaspi* plants was constitutively elevated and the ability to maintain high levels of reduced glutathione seems to be a key factor for both metal tolerance and pathogen resistance [123]. The mentioned above examples prove that plant hormones may play central roles in the tolerance of plants to changing environmental conditions. Moreover, the synergistic or antagonistic hormone actions and the coordinated regulation of hormone biosynthetic pathways in association with the induction of other defence responses, play crucial roles in the adaptation of plants to stresses [124].

Contamination of the environment with heavy metals causes their significant accumulation in organisms at various levels of the food chain [125]. The presence of heavy metals in plants can affect other organisms such as bacteria, fungi and herbivores. As reported by Poschenrieder et al. [15], antagonistic, synergistic or no-effect interactions between metal accumulation and infestation of herbivores or infection caused by pathogenic fungi/pathogenic bacteria may occur. In some cases, heavy metal accumulation in plants may function as a self-defence strategy evolved in hyperaccumulator plants against natural enemies [126]. Metal-hyperaccumulating plants are able to accumulate exceptionally high concentrations of metals in their tissues. A new insight was provided that some metal hyperaccumulating plants can use high concentrations of accumulated metals to defend themselves against attacks by pathogenic microorganisms and herbivores [125]. Already, in the introduction of this review work, we mentioned hypotheses concerning heavy metal-plant-pathogen and heavy metal-plant-insect relationships. The first “Elemental defence” hypothesis presented by Poschenrieder et al. [14] assumes that high metal concentrations in plant tissues are deterrent for herbivores or fungi or can kill these organisms [113]. This hypothesis is based on two modes of action by which the plant may exhibit the “elemental defence” against attacking herbivores: (1) the direct toxicity of the consumed plant material on the attacking herbivore [123,127] and (2) the deterrence of herbivores, whereby plant tissues containing elevated concentrations of elements are less preferred by herbivores than plant material not having such high metal concentrations [113,128,129]. Testing this hypothesis, some studies confirmed the role of the accumulation of Ni [127], Cd [130], Zn [131], As [132] and Se [133] and so forth in plant defence mechanisms. Heavy metals can act against herbivores through their toxic action but this does not safeguard the plant from undergoing damages before poisoning the enemy.

The initial mechanisms enabling insects to avoid heavy metal effects might be “avoidance” [134], which leads them to selectively eat only low-metal tissues of the plant and “dietary dilution,” consisting in lowering overall metal ingestion by eating both high-metal and low-metal containing tissues [129]. On the other hand, experimental evidence does exist that some herbivores prefer to eat low-Zn containing *Thlaspi caerulescens* [110] and low-Ni *Senecio coronatus* [134] when offered a choice between plants containing either low or high-metal concentrations. Deterrent effects have also been observed for Cd [130], As [132] and Se [133]. This ability to avoid feeding on plants with high levels of heavy metals might support that herbivores have a “taste for metals.” Another mechanism deserving a particular interest is “tolerance,” in which physiological adaptations allow specialist herbivores to withstand a high-metal diet, thus disarming the elemental defences of the plant [135]. The bug *Melanotrichus boydi*, for instance, prefers to feed on the Ni hyperaccumulator *Streptanthus polygaloides* [136] and a strain of the moth *Plutella xylostella* (Lepidoptera: Plutellidae) feeds on the Se hyperaccumulator *S. pinnata* without suffering from the high-Se diet [123]. However, since the metal treatment will strongly affect the plants′ metabolome, it might be that herbivores do not directly perceive metals in their food but rather metal-induced metabolites.

The next hypothesis known as “the joint effects,” assumes that many chemicals in plant cell compartments can act separately and/or in combination. If two or more defensive chemicals are enhanced and present combined effects, they display “joint effects” in a defensive model of plant [137]. In general, there are several types of joint effects: additive, synergism and antagonism. The joint effect is considered as additive when two chemicals have a joint effect equal to that of their single effects when combined. The two chemicals display a positive interaction when they have an effect greater than the expected additive effect; the joint effect is thus synergistic. On the contrary, the joint effect is antagonistic if the two chemicals together have an effect lesser than the expected additive effect. “The joint effects” hypothesis has been tested using *Spodoptera exigua* neonates fed with artificial diets [137]. Metal+metal experiments utilized diets amended with metal pairs, using four metals (Co, Cu, Ni and Zn) commonly hyperaccumulated by plants. Metals combined with other metals or organic compounds may be more effective against herbivores than individual metals. The latter authors evaluated the type of joint effects between Co, Cu, Ni and Zn when fed in combinations of two metals to larvae of a model herbivore, *S. exigua* (Lepidoptera: Noctuidae). Boyd and Moar [138] reported that tissues of the Ni hyperaccumulator *S. polygaloides* was toxic to *S. exigua* when plants hyperaccumulated Ni. Cheruiyot et al. [139] used artificial diets to determine lethal and sub lethal concentrations of four metals (Co, Cu, Ni and Zn) to *S. exigua*. Another experiment explored the toxicity of four metals hyperaccumulated by plants (Cd, Ni, Pb and Zn) and searched if metal combinations might broaden the defensive effectiveness of metals in *S. polygaloides* [127]. Metals were used alone and in various combinations such as metal + metal (Zn plus Ni, Pb, or Cd). Artificial diet amended with these treatments was fed to larvae of the crucifer specialist herbivore *Plutella xylostella*. Combinations of metals significantly decreased survival and pupation rates. Effects of combinations were additive rather than synergistic or antagonistic. Because Zn enhanced the toxicity of other metals and Ni enhanced the toxicity of organic defensive chemicals, the defensive effects of metals are more widespread among plants than previously believed.

“The joint effects” hypothesis may justify the simultaneous presence of elemental and organic defences, which may act in concert with each other and enhance the overall plant defences [129]. The results of studies from experiments on inorganic defences and organic defence chemicals in the Ni hyperaccumulator *S. polygaloides* and fed to larvae of *P. xylostella* revealed additive joint effects between Ni and some organic defence chemicals such as atropine, nicotine and tannic acid [127] (Table 1). Combinations of metals plus organic chemicals significantly decreased survival and pupation rates. In turn, the research results of Noret et al. [140] demonstrated that Zn hyperaccumulation in *Thlaspi caerulescens* did not protect plants against snails, *Helix aspersa* (Eupulmonata: Helicidae) and raised the important role of secondary metabolites such as glucosinolates in these plants. Therefore, the above results did not support the defence hypothesis of plants by heavy metals and have shown that nonmetallicolous plants afforded a better protection than metallicolous plants, as glucosinolates increased in plants without Zn after herbivore infestation. Winter et al. [141] discovered that copper (Cu) enhanced the capacity of *Zea mays* to synthesize volatile organic compounds under insect attack. The higher Cu dose was found to be primed for enhanced volatile production that can be triggered by caterpillar feeding. Cu stress correlated with increased levels of ROS in roots and priming of herbivore-induced JA in leaves. In turn, Tolrà et al. [114] demonstrated opposing response of *T. caerulescens* organs after exposure to increasing concentrations of Zn. In leaves of shoots that had accumulated extremely high Zn concentrations, total glucosinolate levels decreased, while in roots the level of glucosinolates increased with increasing Zn in tissues. This mechanism may contribute to increased defence of roots against biotic stressors. In the case of *T. caerulescens* shoot response, the above-mentioned research results support the hypothesis of a trade-off between Zn and glucosinolates.

In turn, “the defensive enhancement” hypothesis suggested that, in some plant species, heavy metals can protect the plants against herbivores and pathogens [137]. Following this hypothesis, the threshold protective benefit concentration is crucial, as each concentration can cause death and/or reduced herbivore growth rate, smaller herbivore size at maturity and reduced fecundity. The increased concentrations of inorganic compounds in herbivore bodies may negatively impact their natural enemies, providing a beneficial component to the herbivore. It has been shown that hyperaccumulation and accumulation of certain elements may affect the defence of plants against herbivores. In the context of this hypothesis, it has been demonstrated that As, Cd, Ni, Se and Zn repelled the herbivores. The research dedicated to providing evidence of the “joint effects hypothesis” has demonstrated defence at relatively low element concentrations and tests of metal/metal and metal/organic compound combinations have revealed joint effects [129]. On the other hand, sub lethal effects of elements may result in more damage to a plant under some scenarios [142]. Heavy metals absorbed by insects have a clear effect on their growth, mortality and physiology [143]. Zn and Cu connect to the cytosol metallothionein in the midgut of many organisms and, as such, are essential elements but at high concentrations they can be toxic [144]. Cd is highly toxic, even at a low concentration and can create mutations in the organism [145]. The effect of heavy metals on an index such as relative growth rate (RGR) is quite various. For example, the effect of a high concentration of Ni in *Spodoptera litura* reduced relative consumption rate (RCR) but a low concentration of Ni increased RGR [146]. A similar study by Baghban et al. [143] on the effect of Cd, Cu and Zn on feeding indices and energy reserves of the cotton boll worm *Helicoverpa armigera* showed that high concentrations of Cd significantly increased approximate digestibility. RGR was significantly enhanced with treatments by Cu, Cd and Zn. It is clear from the present results that the presence of heavy metals in the environment has an intense impact on insects as far as food consumption.

## 4. Effects of Heavy Metals on Insects Including Aphids

Insects play a definite role in the trophic chain and, as food for other organisms, they may constitute an important path for the bioaccumulation of heavy metals. Invertebrates, including insects are good models to study toxicity of heavy metals and, as such, are important bioindicators of environmental contaminations [155]. In the published literature, there were several studies related to the transfer of heavy metals between soil-plant-insect [156]. For example, it has been revealed that the concentration of Cd and Pb declined with increasing trophic levels, while concentrations of Zn and Cu slightly increased from plant to insect. Additionally, numerous literature reports show that the bioamplification (biomagnification) of metals in organisms occurs at higher food chain levels [157,158,159,160]. The presence of heavy metals in insects and its impact on growth rate [161,162,163], mortality [164,165,166,167,168] and physiology [169,170,171] are well-known. Local adaptation of insects to environments contaminated with heavy metals was also reported [172,173,174,175,176]. For example, van Ooik and Rantala [177] demonstrated that the polluted strain (genotype) of the autumnal moth *Epirrita autumnata* displayed a better growth on polluted leaves than the non-polluted strain. Besides, it has previously been found that adding heavy metals to food at moderate levels enhanced the immune system of mots, while a high level of heavy metals decreased their immunity [163]. Moreover, herbivore polyphagic species can avoid heavy metals-containing food [131].

On the other hand, it is known that elements such as Cd are highly toxic, even at a low concentration and have a broad impact on insects causing mutations [145]. Besides, there are some examples of [178,179] harmful effects of this metal on insects. In fact, Kafel et al. [180] demonstrated that the insect *Spodoptera exigua* had a significantly lower survival rate and body weight upon Cd exposure. In turn, the development of *Lymantria dispar* L. larvae in the fourth, fifth and sixth instar was delayed and the larval mass in the third and fourth instar decreased [181]. Additionally, Wu et al. [182] showed that *Boettcherisca peregrina* larvae had altered total larval duration. Moreover, Cd can also have an impact on antioxidant systems in insects [183]. As reported by Zhang et al. [159], some herbivorous insects are strong accumulators of Cd, for example, *Locusta migratoria*, *Oxya chinensis*, *Acrida chinensis* (Orthoptera), *Eligma narcissus* or *Lymantria dispar* (Lepidoptera). As well, Pb ions can affect cells responsible for insect metamorphosis, damage mitochondrial cristae, decrease ATP synthesis and decrease the production of growth hormones [184]. As a result of the influence of Pb, nanism and malformation of the fly imagines *Calliphora vicina*, reduces motor activity, delays the formation and the emergence of puparia [185], reduces the fertility and egg hatching in *Drosophila melanogaster* [186] as well as elongation and finally changes the shape of wings, affect the prolongation and foldation of legs in *Musca domestica* L. [187]. Moreover, there are some research results showing the deleterious effect of Hg on the number of developed oocytes, spermatogonia and spermatocytes in *Culex pipiens* and that Hg causes abnormality of mosquito’s fat body [188]. Also, Hg ions induce oxidative stress in insect species housefly (*Musca domestica*), the cabbage looper moth (*Trichoplusia ni*) [189], two caddisflies (*Chimarra* sp. and *Hydropsyche betteni*) and two mayflies (*Maccaffertium modestum* and *Isonychia* sp.) [190]. Harmful effects on insects have also been identified for other metals such as Mn [178,179], Cr [144], Cu [143,164,191], Hg [164], Zn [143,191,192] and Ni [191]. Hormesis effects of metals on insects are presented in Table 2. It should also be mentioned that the hormesis effect on insects is mostly observed in response to different insecticides [87,193,194,195,196,197,198,199,200,201,202,203].

Plant contamination with heavy metals may directly affect aphids, due to phloem feeding style and relationships with the plant host [204]. Our latest research has revealed the effect of Pb on demographic parameters of pea aphid population, that is, pre-reproductive, reproductive and post-reproductive periods, fecundity and longevity and the feeding process by electronic recording of feeding by the Electrical Penetration Graph technique (EPG) (unpublished data). Moreover, it was shown that contamination with Cu, Pb [205], Zn and Cd [206] in host plants like cabbage (*Brassica oleracea*) and radish (*Raphanus sativus*) affects the developmental instability of the cabbage aphids *Brevicoryne brassicae*. The population of cabbage aphids reared on host plants that accumulated heavy metals showed higher fluctuating asymmetry than population reared on non-contaminated ones. Besides, Cd and Pb effects on morphology of cabbage aphids of both host plants, that is, cabbage and radish, were revealed. All the six measured morphological characters were longer among populations reared on non-contaminated hosts than on contaminated populations, that is, length of ultimate rostral segment, second hind tarsal segment, siphunculi, hind tibia segment, total antennal segment, body length [205]. Moreover, the research of Maryański et al. [207] proved that heavy metal accumulation in aphids might cause additional energy expenditure because of metal detoxification and this might be reflected in an alteration of some traits, such as decreased body mass. These authors showed negative effects of both Cd and Zn on sizes of a number of body parts of carabid beetles, *Poecillus cupreus* L. Moreover, it has been demonstrated that pea aphids are unaffected by Ni accumulation in their host plants such as *Streptanthus polygaloides*. In turn, the aphid *Brachycaudus lychindis* L. feeding on Zn accumulating plants *Silene vulgaris*, can tolerate elevated levels of heavy metals but such an accumulation in turn changes its morphology [208]. The development time of aphids is relatively shorter than for other groups of insects and depends on the host plant. 

Contaminated plants with Cd and Pb separately, have important effects on the life history of aphids. The population characteristics of aphids such as fecundity (number of offspring produced per day) and fitness (intrinsic rate of population increase) were lower on contaminated host plants. However, there was no significant effect on development time (from birth to beginning of the first reproduction). Aphid population reared on the contaminated host plants had significantly decreased reproduction potential and their mortality was about 20% higher on contaminated host plants [209]. Culliney and Pimentel [210] revealed that the green peach aphid *Myzus persicae* fed on collard plants (*Brassica oleracea* L.) growing in pots with soils treated with chemically contaminated sewage sludge, had decreased survival and fecundity rates. In turn, Jaworska and Gospodarek [211] performed experiments with plants that were cultivated in soils contaminated with heavy metals such as Cd, Ni, Cu, Zn and Pb and in soils with natural contents of these elements. It was demonstrated that in both experimental years, aphids preyed more numerously on plants cultivated in the contaminated soil [211]. Besides, Mg fertilization of heavy metal-contaminated soils caused lower Cd and Ni contents and lower the number of *Aphis fabae* [212]. Not all experiments about effects of host plant contaminated with heavy metals on the life history traits are clear and it may depend on many factors. Feeding aphids on contaminated host plants can cause accumulation of harmful metals in their body tissues and affect introduction into food chains [156]. In addition, aphid infestation led to enhanced concentrations of heavy metals in the phloem sap [213]. Studies about the effects of heavy metal- contaminated plants on aphids are limited. Earlier studies also revealed that aphids from contaminated host plants with Cu, firstly excreted Cu with honeydew although its concentration in body tissues did not increase [214]. It has been noted that high metal concentrations had small adverse, not repeatable effects on development, reproduction and growth of aphids. Experiments were planned where green peach aphids (*Myzus persicae* L.) could choose plant *Brassica juncea* contaminated with or without Se. *M. persicae* clearly avoids plants containing Se. In another nonchoice experiment, accumulation of Se decreased up to 50% the growth of aphids and was even lethal. Hanson [154] reported that Se-hyperaccumulators can protect plants from aphid feeding two orders of magnitude higher than the non-hyperaccumulators. Moreover, Boyd and Martens [215] noticed that the concentration of Ni in aphids feeding on high-Ni plants (hyperaccumulating plants) was not dramatically increased. It was found that a high level of Ni in Ni-hyperaccumulator plants did not protect plants against aphids. On the basis of the analysed literature, it can thus be concluded that the influence of metals on aphids is diverse and dependent on heavy metals, plant species and predisposition of the plant for metal accumulation. Therefore, there is the need for further research in this area.

## 5. Effects of Heavy Metals on Fungi

Stimulation of fungal growth by low levels of heavy metals has generally been scarcely reported. In the published literature, there are reports concerning the stimulating effect of heavy metals on the growth of mycelium of pathogenic fungi as well as edible fungi (Table 3). For example, metal ion treatments with 5 mg·L^−1^ of Zn, Cu^2+^ and Fe^2+^ independently had a stimulating effect on the mycelial growth of *Aspergillus flavus* [216]. In turn, salts of Cu^2+^, Fe^2+^ and Fe^3+^ at concentrations such as 5 × 10^−4^ M and 5 × 10^−3^ M enhanced mycelial growth of the fungus *Endothia parasitica*, while at a concentration of 5 × 10^−4^ M, Zn only induced a slight stimulation of the growth of the mycelium [217]. Moreover, the stimulating impact of Pb, at a concentration of 3 ppm, on the mycelial growth of *Pythium debaryanum (R. Hesse*) was observed [218]. Additionally, stimulation of the mycelial growth as a result of the impact of heavy metals such as Zn at concentrations 100–400 mg·kg^−1^ on *Coniothyrum* sp. [219], Cr at 20–100 mg·kg^−1^, Li at 20–100 mg·kg^−1^ and Mn at 10–400 mg·kg^−1^ on *Agrocybe praecox* [220] and Cu at 40 ppm and Pb on *Cryptococcus neoformans* [221] was observed.

Binsadiq and Al-Rahmah [222] demonstrated tolerance mechanisms of fungi in the presence of heavy metals. There are two suggested mechanisms for heavy metal tolerance in fungi, that is, extracellular sequestration (which includes chelation and cell-wall binding) and intracellular sequestration by binding ligands [223]. The influence of heavy metals can exert against microorganisms themselves and through microbial processes [218]. Whipps [224] provided evidence that the influence of heavy metals on the microbial processes in the soil can occur through effects on the decomposition of litter, enzyme activity and growth of plants. In turn, Rudawska et al. [225] showed that heavy metals influence the fungal population by changing the number, the composition and the diversity of microorganisms. The chemical and physical properties of the soil affect the presence of fungi in the soil [226] and these soil characteristics may change according to increased concentrations of heavy metals which can be present separately [227] or in combination [228]. It should be stressed that the interaction between heavy metals and pathogenic fungi is dependent on many factors, that is, the degree of tolerance of the fungi and their possibility of absorption of the heavy metals. However, Agrios [229] indicated that, as a result of adverse environmental factors, different types of fungi became able to survive in the forms of scleroses, chlamydospores, or others. Van West et al. [230] suggested that some fungal species display tolerance to the presence of heavy metals in the environment. In turn, Khan et al. [19] reported that fungi can be used as bioremediators in the polluted soils or as bioindicators. 

On the other hand, the toxic effects of heavy metals on mycelial growth are mostly described in the literature. Stroiński and Floryszak-Wieczorek [231,232] demonstrated that the cell membrane is the first target of the toxic action of metals which can cause electrolyte leakage and modification of the membrane permeability. Besides, heavy metals induce the generation of ROS upon fungal oxidative stress [233]. It must be emphasized that the toxic effects of heavy metals on fungal growth vary widely, depending on the type of metals, their concentration and the considered fungal species [234]. Hartikainen et al. [219,220] studied the effects of Zn, Cu, Cd, Co, Cr and Li on many fungal species belonging to taxons such as Ascomycota, Basidiomycota and Zygomycota. These authors demonstrated harmful effects of these heavy metals on mycelium growth. In addition, Ag, Co, Cu, Fe, Hg, Mn, Pb and Zn had a deleterious impact on fungi of Nematophagus including *Trichoderma harzianum*, *Trichoderma virens*, *Trichoderma hamatum*, *Pochonia chlamydosporia* var. chlamydosporia and *Arthrobotrys oligospora* [235]. Moreover, Abu-Mejdad [221] demonstrated that the ionic forms of Cu and Zn inhibited the mycelial growth of *Aspergilus niger*, *Candida albicans* and *Cryptococcus neoformans*. In the presence of Cu, Zn, Cd and Pb, the mycelial growth speed of the fungi *Fusarium oxysporum* Schlecht. and *Pythium debaryanum* (R. Hesse) was considerably inhibited [218]. 

On the other hand, there are also reports in the published literature showing that the edible fungus *Imleria badia* is capable of accumulating high concentrations of various elements, including toxic metals and metalloids and this fact consequently had a significant effect on the total content of phenolic compounds and the antioxidant potential [236].

Metals can also enhance the resistance of plants to fungal infection. *Triticum aestivum* var Sonalika seedlings pre-exposed to 50 μM CdCl_2_ during 48 h and infected with *Fusarium oxysporum* did not exhibit Fusarium wilt, while the untreated seedlings displayed disease symptoms characteristics for *Fusarium* infection within 7 days post inoculation [148]. Moreover, a similar inhibitory effect of heavy metals such as Cd, Al, Mn and Cu on the development of infections caused by fungal pathogens are presented in Table 3. 

On the other hand, latest research concerning hormesis has focused on the effects of sub lethal doses of metal–containing fungicides on growth and virulence stimulations of plant-pathogenic fungi and oomycetes. There are a lot of reports of hormetic responses in plant pathogens. One of the earliest observations performed by Southam and Ehrlich [237] demonstrated an hormetic response in plant pathogens, for example, stimulation of the metabolism of a wood-decaying fungus *Fomitopsis officinalis* by red cedar extracts. In turn, Hessayon [238] demonstrated the hormesis effect of trichothecin on *Fusarium oxysporum*. Similar examples were also noted in the case of *Pythium aphanidermatum*, which was stimulated by the fungicide mefenoxam [239]. Moreover, the presence of thiabendazole increased the germination of *Penicillium expansum* [240]. Also, the mycelial growths of fungi such as *Lyophyllum palustre* (Peck) Singer [241] and *Phythium aphanidermatum*, *P. irregular* and *P. ultimatum* were increased by propamocarb [242]. Besides, *Phytophthora infestans* growth increased as a result of the impact of metalaxyl [243] and *Sclerotinia sclerotiorum* under treatment with dimethachlon conditions [244].

## 6. Effects of Heavy Metals Deposition in the Environment on the Formation of Ecological Communities of Aphids under Natural Conditions

In recent years, increased interest has arisen in environmental research, whose results have underlined the relationship between the impact of environmental factors, including heavy metals and invertebrates, including aphids in natural uncontrolled conditions. Aphids react to all environmental changes and are considered to be organisms that have the ability to adapt to specific ecological conditions [245]. Abiotic factors such as temperature, photoperiod, atmosphere quality, heavy metals have a significant impact on the development of these insects. Czylok et al. [246] revealed aphid communities occurring in metalliferous areas. Baker et al. [247] reported that ecological communities (biocoenosis) formed on soils with high levels of heavy metals have unique flora and fauna. Adverse environmental factors exert a strong selection pressure on organisms and the appearance, during coevolution, of new ecotypes or kinds of plants which are more resistant [248]. According to Osiadacz and Hałaj [249], it would be important to find out whether the pressure exerted on plants could also affect a higher level of the food chain, that is, primary consumers, especially insects. These authors carried out research to clarify which aphid species and how numerous occur in metalliferous areas and whether they can form characteristic aphid communities. The obtained results demonstrated that metalliferous areas have characteristic communities associated with heavy metal grasslands. They concluded that the high content of metals in the soil did not affect the number of aphid species in the aphid communities but help to form species-rich characteristic for these communities. Moreover, Mackoś–Iwaszko [250] performed observations on the species composition and the number of arthropods from various trophic groups settling the hawthorn *Crataegus xmedia* growing in urban conditions in different degrees of anthropopressure. The formation of proper aphid species in the city plays a crucial role. These works revealed that the sucking-piercing phytophagous aphids were the dominating trophic group settling *C. xmedia*.

In turn, other studies revealed that in an urban environment with different degrees of pollution (street and a large green complex), aphids were abundant on *Acer platanoides* [251]. Besides, the number of aphids was significantly higher in the street site than in a large green complex and the highest number of aphids was observed for *Periphyllus* genus, which are better adapted to urbanized conditions due to high diapausing generations (morphs). The review of Mogren and Trumble [171] evaluated the effects of metal and metalloid pollution on insect behaviors in various environmental conditions. Presented data reveal that a surprising number of insect species cannot detect metal and metalloid contamination, therefore do not always avoid food with significant metal concentrations. The consequence of this was a modification of ingestion, locomotor and reproductive behaviors of insects. Bahadorani and Hilliker and Hanson et al. [154,252] reported that other species of insects moved to contaminated locations, resulting in general reduced species fitness, decrease of population size, species diversity and numerical superiority. In contrast, some species of aquatic insects showed a tendency to escape from contaminated sites [253].

## 7. Conclusions

In the presented review, we have provided data regarding the effect of heavy metals at low doses (the so-called hormetic effect) and toxic doses on the defense responses of plants and the cross-talk of heavy metals and biotic stressors such as insects or pathogenic fungi. Moreover, the hormetic response of insects and pathogens of plants was described. Additionally, we show the relationship between the impact of environmental factors, including heavy metals and invertebrates in natural uncontrolled conditions.

## Figures and Tables

**Table 1:** Effects of heavy metals on the metabolic status of plants and cross talk of heavy metals and biotic stressors.

Metal	Concentration	Fungus/Insect	Plant	Increase of Parameter in Plant	Decrease of Parameter in Plant	References
Al	50 µM AlCl_3_	*Fusarium incarnatum-equiset*	*Cajanus cajan* L.	catalase (CAT), glutathione peroxidase (GPX)	H_2_O_2_, superoxide anion, cell death, superoxide dismutase (SOD), ascorbate peroxidase (APX)	[147]
Al	250 μM AlCl_3_	*Phytophthora infestans* (Mont.)	*Solanum tuberosum* L.	gene expression of: pathogenesis-related (PR) protein 1, PR-2, PR-3, phenylalanine ammonia lyase (PAL), β-1,3 glucanase activity, chitinase activity, H_2_O_2_ salicylic acid (SA), Salicylic acid beta-glucoside (SAG), S-Nitrosothiols (SNOs), fluorescence intensity of nitric oxide (FLNO)—in leaves	SA, SAG, SNOs, FLNO—in roots	[106]
Cd	50 CdCl_2_	*Fusarium oxysporum*	*Triticum aestivum*	Cd^2+^-stress associated protein, free protein thiol content, total protein thiols, glutaredoxin (Grx) acivity	hydrogen peroxide, carbonyl, cysteine content, glutathione (GSH), thiol disulfides,	[148,149]
Cd	1, 10 μM CdCl_2_	*Botrytis cinerea*	*Arabidopsis thaliana*	plant defensin (PDF) 1.2 expression	none	[150]
Cd	250, 500, 750, 1000 mg Cd kg^−1^	*Frankliniella occidentalis*	*Thlaspi caerulescens*	none	leaf feeding damage index (LFDI), number of thrips (*Frankliniella occidentalis*) per plant	[130]
Cu	50 µM CuSO_4_	*Verticillium dahliae* Kleb.	*Capsicum annuum* L.	prolinę oxidase (POX), phenolic compound peroxidase gene (CAPO1), a sesquiterpene cyclase gene (CASC1), a PR1 gene (CABPR1) and a β-1,3-glucanase (CABGLU)	chitinase activity	[107]
Mn	350.0 mg·kg^−1^, MnSO_4_·H_2_O	*Uncinula necator* (Schw.) Burr	*Vitis vinifera*	salicylic acid, abscisic acid (ABA), peroxidase (POD), phenylalanine ammonia lyase (PAL), pathogenesis-related (PR-like) protein, a nucleotide binding site-leucine-rich repeat (NBS-LRR) analogue and a Josephin-like (JOSL) protein	malondialdehyde (MDA), polyphenol oxidase (PPO), SOD, CAT	[151]
Ni	20, 1500 mg·kg^−1^ NiCl_2_	*Spodoptera exigua*	*Streptanthus polygaloides*	larval death	none	[138]
Ni	1820–7960 μg·g^−1^ dry mass	*Melanoplus femurrubrum*, *Evergestis rimosalis*, *Delia radicum*, *Philaenus spumarius*, *Lipaphis erysimi*, *Trialeurodes vaporariorum*, *Lygus lineolaris*, *Tetranychus urticae*	*Streptanthus polygaloides*	none	survival	[127]
Ni	200 µM Ni(NO_3_)_2_	*Erysiphe cruciferarum*	*Thlaspi goesingense*	SA metabolites phenylalanine, cinnamic acid, salicylyl-glucose and catechol	none	[123]
Ni	25, 50, 75 or 100 mg·mL^−1^ Ni	*Pythium mamillatum* and *P. ultimum*	*Alyssum serpyllifolium* ssp. *lusitanicum* and *A. murale*	none	none	[152]
Se	20 μM sodium selenate	*Pieris rapae*, *Alternaria, Brassicicola*, *Fusarium* sp.	*Brassica juncea*	none	feeding rate	[153]
Se	10, 20, 40 μM sodium selenate	*Myzus persicae*	*Brassica juncea*	none	aphids per plant	[154]
Zn	0·5–5 mg·g^−^^1^ ZnSO_4_	*Schistocerca gregaria* *(Forskål)*	*Thlaspi caerulescens* J. & C.	none	time feeding, growth rate, amount eaten	[131]
Zn	10 mg·L^−^^1^ ZnSO_4_·7 H_2_O	*Schistocerca gregaria*, *Deroceras caruanae*, *Pieris brassicae*	*Thlaspi caerulescens* J. & C.	none	preferences	[110]

**Table 2:** Effects of heavy metals at hormetic and toxic doses on invertebrates.

Metal	Concentration	Invertebrata	Hormetic/Toxic Effect	Increase of Parameter in Invertebrata	Decrease of Parameter in Invertebrata	References
Cd	0.0002, 0.00022, 0.0004, 0.0022, 0.0202, 0.2002 ppm CdCl_2_	*Phormia regina* Meig. (Class: Insecta)	hormetic effect	mean percent pupation, stage specific death	mean % emergence, pupae death	[81]
	2.0002, 20.0002, 200.0002 ppm CdCl_2_		toxic effect	pupae death, stage specific death	mean % pupation, mean % emergence,	
Cd	10.53, 7.01, 5.84, 5.25, ng·cm^−2^ Cd^2+^	*Eisenia fetida* (Class: Clitellata)	hormetic effect	catalase (CAT), sodium dismutase (SOD)	none	[159]
	0.33, 0.66 and 1.32 ng·m^−2^ Cd^2+^		toxic effect	none	CAT, SOD	
Cu	0.04 mM, 0.16 mM, 0.63 mM, 2.5 mM, 10 mM, 40 mM and 160 mM, Cu(NO_3_)_2_·3 H_2_O	*Folsomia candida* (Class: Entognatha)	hormetic effect	survival	none	[82]
MeHg	0,2 mM, 0,4 mM MeHgCl	*Caenorhabditis elegans* (Class: Chromadorea)	hormetic effect	expression of glutathione S-transferases (gst-4): GFP (green fluorescence protein)	heat shock proteins (hsp-4):GFP, metallothioneins (mtl-1):GFP and mtl-2:GFP	[84]
Ni	50 and 100 mg·kg^−1^ [Ni(NO_3_)_2_ 6H_2_O]	*Eisenia fetida* (Class: Clitellata)	hormetic effect	microbial biomass carbon, soil basal respiration	dehydrogenase activities	[86]
	300, 500, 800 mg·kg^−1^ [Ni(NO_3_)_2_ 6H_2_O]		toxic effect	none	urease (UA) and dehydrogenase activities	
U	1.86, 5.0, 9.3 mg·kg^−1^ depleted uranium (DU)	*Eisenia fetida* (Class: Clitellata)	hormetic effect	natural red retention time, DNA breaks	toxicity factor	[83]
	18.6, 50, 93, 150, 186, 300 and 600 mg·kg^−1^ DU		toxic effect	DNA breaks	toxicity factor	

**Table 3:** Effects of heavy metals at hormetic and toxic dose on fungi.

Metal	Concentration	Plant Species	Hormetic/Toxic Effect	Increase of Parameter in Fungi	Decrease of Parameter in Fungi	References
Cd	5 mg·L^−1^	*Aspergillus flavus*	hormetic effect	total RNA, aflatoxin, O-methylsterigmatocystin	none	[216]
Cr	20–100 mg·kg^−1^ KCr(SO_4_)_2_·12 H_2_O	*Agrocybe praecox*	hormesis effect	none	Enzyme production	[220]
Cr	20–100 mg·kg^−1^ KCr(SO_4_)_2_ ·12 H_2_O	*Pleurotus pulmonarius*, *Phlebia radiata*, *Physisporinus rivulosus* and *Stropharia rugosoannulata*	toxic effect	none	none	
Cu	5 mg·L^−1^	*Aspergillus flavus*	hormetic effect	total RNA, aflatoxin, O-methylsterigmatocystin	none	[216]
Cu	5·10^−4^ M, 5·10^−3^ M CuSO_4_ and CuCl	*Endothia parasitica*	hormetic effect	none	none	[217]
Cu	3 ppm CuSO_4_·5H_2_O	*Suillus luteus*	hormetic effect	none	none	[218]
Cu	20 ppm	*Cryptococcus neoformans*	hormetic effect			[221]
	40 ppm		toxic effect			
Fe	5 mg·L^−1^	*Aspergillus flavus*	hormetic effect	total RNA, aflatoxin, O-methylsterigmatocystin	none	[216]
	5·10^−4^ M and 5·10^−3^ M FeC1_2_·4H_2_O. FeC1_3_·6H_2_O	*Endothia parasitica*	hormetic effect	none	none	[217]
Li	20–100 mg·kg^−1^ Li_2_ SO_4_·H_2_O	*Agrocybe praecox*	hormetic effect	none	none	[220]
	20–100 mg·kg^−1^ Li_2_ SO_4_·H_2_O	*Pleurotus pulmonarius*, *Phlebia radiata*, *Physisporinus rivulosus* and *Stropharia rugosoannulata*	toxic effect	none	none	
Pb	3, 33 ppm Pb(NO_3_)_2_	*Suillus luteus*	hormetic effect	none	none	[218]
Pb	3 ppm Pb(NO_3_)_2_	*Hebeloma* spp.	hormetic effect	none	none	
Mn	10–400 mg·kg^−1^ MnSO_4_·4H_2_O	*Agrocybe praecox*	hormetic effect	none	none	[220]
Mn	10–400 mg·kg^−1^ MnSO_4_·4 H_2_O	*Pleurotus pulmonarius*, *Phlebia radiata*, *Physisporinus rivulosus* and *Stropharia rugosoannulata*	toxic effect	none	none	
Zn	5·10^−4^ M ZnSO_4_·7H_2_O	*Endothia parasitica*	hormetic effect	none	none	[217]
Zn	3 ppm ZnSO_4_·7H_2_O	*Hebeloma* spp.	hormetic effect	none	none	[218]
Zn	100, 200, 400 mg Zn kg^−1^	*Coniothyrium* sp.	hormetic effect	none	enzyme production on ABTS malt extract agar plates	[219]
	100 mg·kg^−1^ Zn	*Agrocybe praecox*, *Gymnopus peronatus*, *Gymnopilus sapineus*, *Stropharia rugosoannulata*, *Mycena galericulata*		none	none	
	100, 200, 400 mg Zn kg^−1^	*Sordaria* sp., *Pyrenophora* sp., *Alternaria* sp., *Chaetomium* sp., *Fusarium* sp., *Epicoccum* sp., *Gliocladium* sp., *Mortierella* sp., *Cylindrocarpon* sp.	toxic effect	Enzyme production on ABTS malt extract agar plates	none	
	100 mg·kg^−1^ Zn	*Agaricus bisporus*, *Gymnopilus luteofolius*, *Stropharia aeruginosa*	toxic effect	none	none	

## Supplementary Materials

Supplementary materials (Table S1) can be found online.
