# Supplementary material for: The Role of Heavy Metals in Plant Response to Biotic Stress
Source: Molecules. 2018 Sep 11;23(9):2320. doi: 10.3390/molecules23092320 (PMC6225295; doi:10.3390/molecules23092320)
Supplement: Supplementary file 1 [file molecules-23-02320-s001.pdf]

Table S1. Effects of heavy metals on hormetic and toxic doses on physiological and biochemical factors in plants

Metal	Plant species	Plant organ	Hormetic/ toxic effect	Concentration	Increase of parameter	Decrease of parameter	Authors
Al	*Oryza sativa* L.	shoot, root	hormetic effect	200 μM $AlCl_3$	Chlorophyll a and b, total chlorophyll, free amino acids – Cotaxtla, Huimanguilo, Temporalero cultivars; soluble sugars, P, K – in roots; N - Cotaxtla cultivar in roots; Ca – Huimanguilo cultivar in roots; Mg - Cotaxtla, Tres Rios, Huimanguilo cultivars in roots; N, Mg - in shoots; K - in Cotaxtla, Tres Rios, Huimanguilo cultivar in shoots; Ca - in Tres Rios, Temporalero cultivars in shoots	Chlorophyll a and b, total chlorophyll, free amino acids - Tres Rios cultivar, proline; N - in Cotaxtla, Tres Rios, Huimanguilo cultivars in roots; Ca - in Cotaxtla, Tres Rios, Temporalero cultivar in roots; Mg - in Temporalero cultivar in roots; P - in shoots; K - in Temporalero cultivar in shoots; Ca - in Cotaxtla, Huimanguilo cultivar in shoots	[44]
Al	*Camellia sinensis* L.	root	hormetic effect	400 μM$AlCl_3$	superoxide dismutase (SOD), catalase (CAT), ascorbate peroxidase (APX)	Lipid peroxidation, wall-bound phenols, lignin, phenylalanine ammonia lyase (PAL), soluble fraction of proline oxidase (POX), ionically wall bound peroxidases, covalently wall bound peroxidases	[53]
Al	*Melastoma malabathricum L.*	root, stem, leaf	hormetic effect	0.5 mM $AlCl_3$	N, P - in young, mature and old leaves; K - in mature and old leaves	K - in young leaves; N, K – in roots	[54]
Al	*Glycine max* (L.) Merr.	root	hormesis effect	25 μM $AlCl_3·6H_2O$	SOD, peroxidase (POD), malondialdehyde (MDA) content	-	[55]
			toxic effect	50 μM $AlCl_3·6H_2O$	SOD, POD, MDA content	-	

Al	*Zea mays* L.	leaf	hormesis effect	48 µM $AlCl_3{\cdot}7H_2O$	MDA content, superoxide radicals (SOR) production, protein, chlorophylls	SOD, POD, CAT, K, Ca, Mg, photosynthetic rate	[56]
As	*Spirodela polyrrhiza* L.	seedling	hormesis effect	1, 5, 10 µM Na_2HAsO_4	Total protein content - 1 and 4 days after exposure; chlorophyll a, b and t, carotenoids - 1 and 4 days after exposure; non protein thiols, cystein content, ascorbic acid content - 1 and 4 days after exposure	Total protein content - 7 days after exposure; chlorophyll a, b and t, carotenoids - 7 days after exposure; non protein thiols, cystein content, ascorbic acid content - 7 days after exposure	[65]
			toxic effect	20 µM Na_2HAsO_4	Total protein content - 1day after exposure; chlorophylls a, b and t - 1 day after exposure; carotenoids - 1 and 4 days after exposure; non protein thiols, cystein content, ascorbic acid content - 1 and 4 days after exposure	Total protein content - 4 and 7 days after exposure; chlorophylls a, b and t - 4 and 7 days after exposure; carotenoids - 7 days after exposure; non protein thiols, cystein content, ascorbic acid content - 7 days after exposure	
As	*Pteris vittata* L.	fern	hormesis effect	100 ppm chromated copper arsenate (CCA)	-	-	[254]
Cd	*Gypsophila fastigiata* L.	shoot	hormesis effect	0.5, 2.5, 5 µM $CdCl_2$.	total phenols phenylpropanoids, flavonols anthocyanins	Chlorophylls a and b, carotenoids	[60]

Cd	*Sedum alfredii* Hance	shoot	hormesis effect	25, 50, 100 μmol·L^{-1} $CdCl_2$	K, Ca, Mg, P, Zn- in leaves<br>Cu – in leaves for 25 μmol· L^{-1} $CdCl_2$<br><br>P, Cu, B – in stems<br>K, Mg, S – in stems for 50 and 100 μmol· L^{-1} $CdCl_2$<br>Fe – in stems for 25 and 100 μmol· L^{-1} $CdCl_2$<br>Zn – in stems for 25 and 50 μmol· L^{-1} $CdCl_2$	S, Fe, Mn – in leaves<br>Cu and B – in leaves for 50 and 100 μmol· L^{-1} $CdCl_2$<br><br>K, Ca, Mg, S – in stems for 25 μmol· L^{-1} $CdCl_2$<br>Fe – in stems for 50 μmol· L^{-1} $CdCl_2$<br>Zn – in stems for 100 μmol· L^{-1} $CdCl_2$<br>Mn – in stems	[57]
			toxic effect	200, 400, 800 μmol·L^{-1} $CdCl_2$	K, Ca, Mg, P, Fe- in leaves for 200 and 400 μmol·L^{-1} $CdCl_2$<br>S – in leaves only for 200 μmol·L^{-1} $CdCl_2$<br><br>Zn, Fe – in stems<br>K, P, S, Cu – in stems only for 200 and 400 μmol·L^{-1} $CdCl_2$;<br>B – in stems only for 400 and 800 μmol·L^{-1} $CdCl_2$;<br>Mn - in stems only for 400 μmol·L^{-1} $CdCl_2$	K, Mg, P, Fe – in leaves only for 800 μmol·L^{-1} $CdCl_2$<br>Mn, Cu, B – leaves<br>S – in leaves only for 400 and 800 μmol·L^{-1} $CdCl_2$<br><br>Ca, Mg – in stems;<br>Mn - stems only for 200 and 400 μmol·L^{-1} $CdCl_2$<br>P, S – in stems only for 800 μmol·L^{-1} $CdCl_2$<br>B – in stems only for 200 μmol·L^{-1} $CdCl_2$	
Cd	*Viola baoshanensis* W.S.Shu, W.Liu & C.Y.Lan	plant (no distinction)	hormesis effect	5, 10, 20, 30 mg·L^{-1} $CdCl_2$	-		[58]
			toxic effect	40, 50, 60 mg·L^{-1} $CdCl_2$			

Cd	*Lonicera japonica* Thunb.	root, stem, leaf	hormetic effect	5, 10 mg kg^{-1} $CdCl_2 \cdot 2.5\ H_2O$	Net photosynthesis rate, stomatal conductance;<br>transpiration rate - only for 5 mg kg^{-1} $CdCl_2 \cdot 2.5H_2O$, photochemical efficiency of Photosystem II (PSII), effective quantum yield of PSII, phytochemical quenching coefficient, potential efficiency of PSII phytochemistry, chlorophyll a, chlorophyll b, carotenoids, total chlorophylls	Intercellular CO_2 concentration;<br>transpiration rate - only for 10 mg kg^{-1} $CdCl_2 \cdot 2.5H_2O$	[59]
			toxic effect	25, 50, 100 $CdCl_2 \cdot 2.5\ H_2O$	Phytochemical quenching coefficient - only for 25 $CdCl_2 \cdot 2.5H_2O$;<br>carotenoid - only for 50, 100 $CdCl_2 \cdot 2.5H_2O$	Net photosynthesis rate, stomatal conductance, intercellular CO_2 concentration, photochemical, transpiration rate, efficiency of PSII, effective quantum yield of PSII, phytochemical quenching coefficient, potential efficiency of PSII phytochemistry; chlorophylls a and b, carotenoids - only for 100 $CdCl_2 \cdot 2.5H_2O$;<br>totall chlorophyls	
Cd	*Dianthus carhusianorum* L.	shoot, root	hormetic effect	1, 3, 5.5 μM $CdCl_2$	K, Fe;<br>Zn, chlorophyll a and b, carotenoids - only for 3 and 5.5 μM $CdCl_2$	Na, Mg, Ca, Mn, Cu, total phenols, phenylpropanoids;<br>Zn, chlorophyll a and b, carotenoids, flavonols, anthocyanins - only for 3 and 5.5 μM $CdCl_2$	[60]
Cd	*Solanum melongena* L.	shoot, root	hormetic effect	10^{-8} M $CdCl_2$	-	protochlorophylls, chlorophylls a and b, total chlorophylls	[61]

			toxic effect	10^{-2}, 10^{-4}, 10^{-5} M $CdCl_2$	-	protochlorophylls, chlorophylls a and b, total chlorophylls (strong decrease)	
Cd	*Arabis paniculata* F.	seedling	hormetic effect	22, 44, 89 μM $CdCl_2$	Chlorophylls a/b;<br>MDA – in leaves for 44 μM $CdCl_2$<br>superoxide anion, SOD, CAT, glutathione peroxidase (GPX), ascorbate peroxidase (APX) – in leaves<br>glutathione reductase (GR)– in leaves only for 89 μM $CdCl_2$	chlorophylls a and b, carotenoids, total chlorophylls- in leaves<br>MDA – in leaves only for 22 and 89 μM $CdCl_2$<br>hydrogen peoxide;<br>GR – in leaves only for 89 μM $CdCl_2$	[62]
					CAT – in root only for 22, 44 μM $CdCl_2$	MDA, hydrogen peroxide, superoxide anion, SOD, GPX, APX, GR - in root;<br>CAT - in root only for 89 μM $CdCl_2$	
			toxic effect	178 μM $CdCl_2$	MDA, hydrogen peroxide, SOD, CAT, GPX, APX, GR – in leaves<br>GR – in roots	superoxide anion – in leaves<br>MDA, hydrogen peroxide, superoxide anion, SOD, CAT, GPX, APX – in roots	
Cd	*Brassica juncea* L.	root, shoot	hormetic effect	10, 20, 40, 80, 160 mM $CdCl_2 \cdot H_2O$ - 14 days after exposure	Chlorophylls a, b and t, carotenoids non protein thiols, gluthation, total protein content phytochelatin content, GR activity (strong increase)	-	[63]
			toxic effect	10, 20, 40, 80, 160 mM $CdCl_2 \cdot H_2O$ - 28 days after exposure	Chlorophylls a, b and t, carotenoids, non -protein thiols, gluthation, total protein content phytochelatin content, GR activity	-	

Cd	*Salsola kali* L.	root	hormetic effect	20 mg L^{-1} of Cd(II)	Ca, Mn, Mo	K, P, Mg, Fe, Cu, Zn	[64]
			toxic effect	5, 10 mg L^{-1} of Cd(II)	K, P, Ca, Cu	Mg, Fe, Zn, Mn, Mo	
Cd	*Spirodela polyrrhiza* L.	seedling	hormesis effect	0.1, 0.5, 1 μM $CdCl_2{\cdot}H_2O$	Total protein content - 1 day after exposure; chlorophylls a, b and t - 1 day after exposure and for 0.1,0.5 μM Cd^{2+} 4 day after exposure; carotenoids, non-protein thiols, cysteine content - 1 and 4 days after exposure; ascorbic acid -1 day after exposure	Total protein content - 4 and 7 days after exposure; chlorophylls a, b and t - 7 days after exposure and for 1 μM Cd^{2+} 4 days after exposure; carotenoids, non-protein thiols, cysteine content - 7 days after exposure; ascorbic acid – 4 and 7 days after exposure	[65]
			toxic effect	2 μM $CdCl_2{\cdot}H_2O$	Total protein content, chlorophylls a, b and t, ascorbic acid - 1 day after exposure; carotenoids, non protein-thiols , cystein content - 1 and 4 days after exposure	Total protein content, chlorophylls a, b and t, ascorbic acid - 4 and 7 days after exposure; carotenoids, non-proteic thiols, cystein content - 7 days after exposure	
Cd	*Arabis paniculata* Franch	root, shoot	hormetic effect	9, 44, 89, 178 μM Cd $CdCl_2{\cdot}2.5H_2O$	chlorophylls a and b, chlorophylls a+b	-	[66]
			toxic effect	267 μM Cd $CdCl_2{\cdot}2.5H_2O$	chlorophyll a, chlorophylls a+b	chlorophyll b	
Cr	*Eichhornia crassipes* Mart.	root, aerial parts	hormetic effect	2.0, 4.0 mg·L^{-1} $CrCl_3{\cdot}6H_2O$	chlorophylls a and b, carotenoids; CAT, POD, SOD, GR activity, MDA - in roots and aerial parts	CAT activity - in aerial parts 72 h after exposure	[67]
			toxic effect	6.0, 8.0 mg·L^{-1} $CrCl_3{\cdot}6H_2O$	chlorophylls a and b, carotenoids, CAT, POD, SOD, GR acivity, MDA - in roots and aerial parts	CAT activity – only in aerial parts 72 h after exposure	

Cr	*Salsola kali* L.	root, shoot	hormetic effect	5, 10 mg·L^{-1} $K_2Cr_2O_7$ – in roots	Ca and Mg, Cu Zn - for 5 mg·L^{-1} Cr(VI)	K, P, Fe and Mg, Cu, Zn - for 10 mg·L^{-1} Cr(VI)	[68]
				5, 10 $Cr(NO_3)_3$ – in roots	Ca- for 10 $Cr(NO_3)_3)$; Fe - for 5 $Cr(NO_3)_3)$	K, P, Mg, Cu, Zn;<br>Ca, Fe - for 5 $Cr(NO_3)_3)$	
				5, 10 mg·L^{-1} $K_2Cr_2O_7$ – in shoots	K, Fe – in stem for 10 mg·L^{-1} $K_2Cr_2O_7$<br>-	Ca, P, Mg, Cu, Zn and K, Fe – in stem for 5 10 mg·L^{-1} $K_2Cr_2O_7$<br>Ca, K, P, Mg, Fe, Cu, Zn – in leaves	
				5 mg·L^{-1} $Cr(NO_3)_3$ – in shoots	K;<br>Fe - in stem only for 10 mg·L^{-1} $Cr(NO_3)_3$<br>Zn;<br>K, P – in leaves only for 10 mg·L^{-1} $Cr(NO_3)_3$	Ca, P, Mg, Cu, Zn; Fe – in stem only for 5 mg·L^{-1} $Cr(NO_3)_3$<br>Ca, Mg, Fe, Cu;<br>K, P – in leaves for 5 $Cr(NO_3)_3$	
			toxic effect	20 mg·L^{-1} $K_2Cr_2O_7$ – in roots	Ca	K, P, Mg, Fe, Cu, Zn	
				20 mg·L^{-1} $Cr(NO_3)_3$ – in roots	Ca	K, P, Mg, Fe, Cu, Zn	
				20 mg·L^{-1} $K_2Cr_2O_7$ – in shoot	K - in stem<br>Zn – in leaves	Ca, P, Mg, Fe, Cu, Zn – in stem<br>Ca, K, P, Mg, Fe, Cu – in leaves	
				10, 20 mg·L^{-1} $Cr(NO_3)_3$ – in shoots	Fe – in stem<br>K – in leaves	Ca, K, P, Mg, Cu, Zn – in stem<br>Ca, P, Mg, Fe, Cu, Zn – in leaves	
Cr	*Lemna minuta* Kunth		hormesis effect	2.5, 5 mg·L^{-1} $Cr_2O_4K_2$	No differences	chlorophylls, carotene and malondialdehyde	[69]

			toxic effect	10, 20 mg·L^{-1} $Cr_2O_4K_2$			
Cr	*Allium cepa L.*	root	hormesis effect	12.5 μM CrO_3	CAT, GPX, SOD, APX activity deoxyribonucleic acid (DNA) damage	superoxide radical, hydrogen peroxide, hydroxyl radical, cell death, lipid peroxidation	[70]
			toxic effect	25, 50, 100, 200 μM CrO_3	superoxide radical, hydrogen peroxide, hydroxyl radical, cell death, lipid peroxidation, GPX activity, DNA damage	CAT, SOD, APX acivity	
La	*Capsicium annum* L.	shoot, leaf	hormetic effect	10 μM $LaCl_3$	total soluble sugars total soluble proteins chlorophylls a and b, total chlorophylls	total soluble amino acids	[71]
La	*Lonicera japonica* Thunb.	leaf	hormetic effect	10, 20, 30 mg·L^{-1} (La III)	POD activity, chlorophyll content, chlorophylls a/b value	relative electrolyte leakage, MDA, H_2O_2 content	[72]
			toxic effect	60, 100 mg·L^{-1} (La III)	relative electrolyte leakage, MDA, H_2O_2 content	POD activity, chlorophyll content, chlorophylls a/b value	
La	*Oryza sativa* L.	root	hormetic effect	0.05, 0.1 mmol·L^{-1} $La(NO_3)_3$	K, Mg, Ca, Na, Mn, Mo	Fe, Zn, Cu	[73]
			toxic effect	0.5, 1, 1.5 mmol·L^{-1} $La(NO_3)_3$	Mg, Na, Fe, Mn	K, Ca, Zn, Cu, Mo	
Pb	*Arabis paniculata* Franch	roots, shoot	hormesis effect	24, 48 μM $Pb(NO_3)_2$	chlorophyll a; chlorophylls a+b - for 24 μM $Pb(NO_3)_2$	chlorophyll b; chlorophylls a+b - for 48 μM $Pb(NO_3)_2$	[66]

			toxic effect	97, 193, 386 μM $Pb(NO_3)_2$	chlorophyll a, chlorophylls a+b - for 97 μM $Pb(NO_3)_2$	Chlorophyll b; Chlorophyll a and chlorophylls a+b - for 193, 386 μM $Pb(NO_3)_2$	
Pb	*Pisum sativum* L.	shoot	hormesis effect	0.075 mM $Pb(NO_3)_2$	TSA, SA, SAG, ABA, pisatin, 2'OH genistein, Glc-Glc-Glc rhamnose, PAL	Glc-Glc-Glc kaempferol, β-glucosidase	[35]
			toxic effect	0.5 mM $Pb(NO_3)_2$	TSA, SA, SAG, ABA, pisatin, 2'OH genistein, Glc-Glc-Glc rhamnose, PAL	Glc-Glc-Glc kaempferol, β-glucosydase	
Pb	*Brassica pekinensis* Rupr.	root, shoot	hormesis effect	125, 250, 500, 1000 $\mu g \cdot mL^{-1}$ $Pb(NO_3)_2$	-		[74]
Pb	*Dianthus carthusianorum* L.	shoot	hormesis effect	0.1, 0.5, 1 mM $Pb(NO_3)_2$	Zn, Fe; Cu - for 0.1, 0.5 mM $Pb(NO_3)_2$ Mg, K - for 1 mM $Pb(NO_3)_2$	Na, Ca, Mn; Mg, K – only for. 1 mM $Pb(NO_3)_2$; Cu – only for 1 mM $Pb(NO_3)_2$	[60]
Pb	*Anthyllis vulneraria* L.	shoot	hormesis effect	0.5, 1 mM $Pb(NO_3)_2$	Phenols, anthocyanins, MDA, POD, CAT, chlorophylls a and b, carotenoids	-	[75]
			toxic effect	1.5 mM $Pb(NO_3)_2$	Phenols, anthocyanins, MDA, POD, chlorophylls a and b, carotenoids	CAT	
Hg	*Helianthus tuberosus* L.	stem	hormesis effect	0.15, 1 mg kg^{-1} $HgCl_2$	MDA - for 1 mg kg^{-1} $HgCl_2$; Pn, chlorophylls - for 0.15 mg kg^{-1} $HgCl_2$	Pn, chlorophyll, Fv/Fm - for 1 mg kg^{-1} $HgCl_2$	[76]
			toxic effect	5, 10 mg kg^{-1} $HgCl_2$	MDA; Pn - for 10 mg kg^{-1} $HgCl_2$	Pn - for 5 mg kg^{-1} $HgCl_2$; chlorophylls, Fv/Fm	
Pd	*Pisum sativum* L.	root	hormesis effect	0.10, 0.25, 1.0, mg L^{-1}	Photosynthetic efficiency	-	[77]

				K_2PdCl_4			
			toxic effect	0.10, 0.25, 10, 25 mg L^{-1} K_2PdCl_4	Photosynthetic efficiency	-	
Pt	*Arabidopsis thaliana* L.	root, rosette	hormetic effect	2.5 μM $Pt(NH_3)_4](NO_3)_2$	net photosynthesis<br>stomatal resistance<br>chlorophyll content<br>glutathione, phytochelatins	-	[78]
			toxic effect	5, 25, 50, 100 μM. $Pt(NH_3)_4](NO_3)_2$	glutathione, phytochelatins	net photosynthesis<br>stomatal resistance<br>chlorophyll content	
Ti	*Avena sativa* L.		toxic effect	2 ppm $TiCl_4$	-	-	[255]
U	*Schizachyrium scoparium* Michx.	root, shoot	hormesis effect	50, 500 mg $kg^{\cdot -1}$ $UO_2(OH)_2$-nH2O	-	-	[79]
			toxic effect	500, 25000 mg $kg^{\cdot -1}$ $UO_2(OH)_2$-nH2O			
Zn	*Thlaspi caerulescens* J.Presl & C.Presl	shoot	hormesis effect	1000, 2000 $\mu g \cdot g^{-1}$ ZnSO4	Mg	K, Ca, P, Cl	[80]
			toxic effect	3000, 4000 $\mu g \cdot g^{-1}$ ZnSO4	-	Mg, K, Ca, P, Cl	
Zn	*Sedum alfredii* Hance	root, shoot	hormesis effect	5, 10, 20, 40, 80, 160 $mg \cdot L^{-1}$ $ZnSO_4 \cdot 7\ H_2O$ - in shoots<br>20, 40, 80, 160 $mg \cdot L^{-1}$ $ZnSO_4 \cdot 7\ H_2O$ – in roots	-	-	[57]
			toxic effect	240, 320 $mg \cdot L^{-1}$ $ZnSO_4 \cdot 7\ H_2O$			

Zn	*Arabis paniculata* Franch	root, shoot	hormesis effect	306, 612, 1223; 2447 μM $ZnSO_4 \cdot 7H_2O$ - in shoots ; 153, 306 μM $ZnSO_4 \cdot 7H_2O$ - in roots	Chlorophylls a and b, chlorophylls a+b	-	[66]
			toxic effect	1223, 2447 μM $ZnSO_4 \cdot 7 H_2O$ – in roots	Chlorophyll b, chlorophylls a+b; Chlorophyll a – for 1223 μM $ZnSO_4 \cdot 7 H_2O$	Chlorophyll a – for 2447 μM $ZnSO_4 \cdot 7 H_2O$	
